# EDLDR: An Ensemble Deep Learning Technique for Detection and Classification of Diabetic Retinopathy

**DOI:** 10.3390/diagnostics13010124

**Published:** 2022-12-30

**Authors:** Sambit S. Mondal, Nirupama Mandal, Krishna Kant Singh, Akansha Singh, Ivan Izonin

**Affiliations:** 1 Department of Electronics & Communication Engineering, Asansol Engineering College, Asansol 713305, India; 2 Department of Electronics Engineering, IIT (ISM) Dhanbad, Dhanbad 826004, India; 3 Department of CSE, ASET, Amity University, Noida 201301, India; 4 School of CSET, Bennett University, Greater Noida 201310, India; 5 Department of Artificial Intelligence, Lviv Polytechnic National University, 79000 Lviv, Ukraine

**Keywords:** diabetic retinopathy, DenseNet, ResNeXt, ensembling

## Abstract

Diabetic retinopathy (DR) is an ophthalmological disease that causes damage in the blood vessels of the eye. DR causes clotting, lesions or haemorrhage in the light-sensitive region of the retina. Person suffering from DR face loss of vision due to the formation of exudates or lesions in the retina. The detection of DR is critical to the successful treatment of patients suffering from DR. The retinal fundus images may be used for the detection of abnormalities leading to DR. In this paper, an automated ensemble deep learning model is proposed for the detection and classification of DR. The ensembling of a deep learning model enables better predictions and achieves better performance than any single contributing model. Two deep learning models, namely modified DenseNet101 and ResNeXt, are ensembled for the detection of diabetic retinopathy. The ResNeXt model is an improvement over the existing ResNet models. The model includes a shortcut from the previous block to next block, stacking layers and adapting split–transform–merge strategy. The model has a cardinality parameter that specifies the number of transformations. The DenseNet model gives better feature use efficiency as the dense blocks perform concatenation. The ensembling of these two models is performed using normalization over the classes followed by maximum a posteriori over the class outputs to compute the final class label. The experiments are conducted on two datasets APTOS19 and DIARETDB1. The classifications are carried out for both two classes and five classes. The images are pre-processed using CLAHE method for histogram equalization. The dataset has a high-class imbalance and the images of the non-proliferative type are very low, therefore, GAN-based augmentation technique is used for data augmentation. The results obtained from the proposed method are compared with other existing methods. The comparison shows that the proposed method has higher accuracy, precision and recall for both two classes and five classes. The proposed method has an accuracy of 86.08 for five classes and 96.98% for two classes. The precision and recall for two classes are 0.97. For five classes also, the precision and recall are high, i.e., 0.76 and 0.82, respectively.

## 1. Introduction

**Figure 1:**
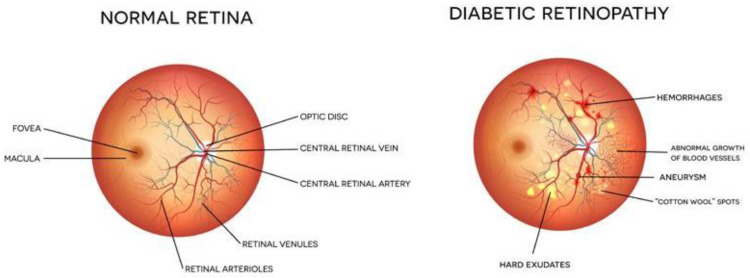
Eye structure and presence of DR [Image credit https://www.eyeops.com/, accessed on 20 September 2022].

Diabetes is a chronic disease that occurs either when the pancreas does not produce enough insulin or when the body cannot effectively use the insulin it produces. In this case, the glucose level in the blood becomes high, leading to multiple problems. It is reported that about 10% of the total world’s population is suffering from diabetes (WHO). This number is increasing rapidly and is expected to increase manifold in the coming years. Diabetes affects other body organs and systems. Specifically, the nerves and the blood vessels are most affected. The vision of diabetic persons is often affected due to diabetic retinopathy (DR). This is an eye condition that leads to vision loss and blindness. The blood vessels of the retina are damaged leading to the formation of exudates. These exudates lead to a loss in the vision of the affected person. In the early stages of DR, the symptoms may go unnoticed or be misinterpreted. A few patients may feel a minor alteration in their vision, for example, difficulty in reading or seeing objects that are far away. However, in the advanced stages of DR, the blood vessels of the retina start bleeding and, thus, a gel-like fluid fills up the eyes. Due to this, spots or streaks appear in the eyes, leading to loss of vision. There are two main stages of diabetic retinopathy, known as non-proliferative and proliferative diabetic retinopathy. The early stage of DR is known as non-proliferative diabetic retinopathy (NPDR). In this stage, tiny blood vessels leak, leading to swelling in the retina. In case of NPDR, the vision becomes blurry due to the presence of exudates. The advanced stage is known as proliferative diabetic retinopathy (PDR). In this condition, the blood vessels bleed. If the bleeding is high, the vision becomes blocked. Figure 1 shows the images of a normal eye and an eye affected with DR.

The early detection of DR in patients helps in their treatment. Thus, various automated tools and advanced techniques are available in the literature for automated detection of DR from retinal images. In this paper, a deep learning-based approach is proposed for the detection and classification of DR from retinal images. In the next section, a literature review of the existing techniques for exudate detection is discussed.

## 2. Literature Review

Detection of diabetic retinopathy is critical in the treatment of patients. Presently, a trained ophthalmologist performs manual analysis of the fundus images to detect the presence of DR. This method requires the availability of trained individual and is prone to human errors. Thus, there is a need for automated methods that can analyse fundus images. The automated methods are fast and accurate. In the literature, many methods based on image processing and machine learning are available for DR detection and classification. Many researchers have used segmentation techniques for the identification of exudates and discs. Maximum principal curvature is used for the segmentation of exudates or any other abnormality in the eye [1]. Another author presented a blood vessel segmentation technique for analysis of the retinal image vessels. Image morphological operators along with K-means clustering are utilized for the segmentation of blood vessels [2]. Many other authors have proposed different image segmentation-based techniques for detection of DR [3,4,5]. The literature review reveals that mostly Gaussian methods [6], mathematical morphology [7] and multi-scale analysis [8] are being widely used for detection of DR. All these methods based on image processing do not show very high accuracy for the detection of DR. Thus, more efficient methods are required that can identify and classify the retinal images more accurately. Nowadays, deep learning is being widely used for image classification problems. Many researchers have made use of various deep learning models for detection and classification of DR [8]. Convolutional neural networks have been widely used for DR detection and classification [9,10,11]. The performance of these networks was further improved using transfer learning along with CNN [12]. With transfer learning, the pretrained models can be tuned as per the medical image database consisting of retinal images. These models demonstrated improved accuracy as compared to the traditional CNN [13,14]. Many researchers proposed ensemble methods that combined the advantages from different classifiers and produced highly accurate results. The ensemble models have a higher information gain, as the information from standalone models is combined [15]. There are many ensembling techniques that can be used for the combining of complementary information amongst models. Many ensemble classifiers are reported in the literature that are presented for the DR problem [15,16,17,18,19]. The literature review reveals that there are certain limitations in the existing methods. Due to a limited database of medical images, the accuracy of machine learning models is not high. In addition, the traditional methods have the efficiency of a single model. However, the proposed method performs augmentation; thus, the model is immune to orientation variations.

In this paper, to further improve the accuracy and speed of DR detection and classification DRRest is proposed. DRRest is a deep learning ensemble model that uses ResNext architecture. The ResNeXt architecture comprises a shortcut from the previous block to the next block, stacking layers and adapting split–transform–merge strategy. The model has a cardinality parameter that specifies the number of transformations. The model is trained on the third largest dataset APTOS19 which includes more than 5000 retinal images. The images are pre-processed using CLAHE method for histogram equalization. The dataset has a high-class imbalance, and the images of the non-proliferative type are very low, therefore, GAN-based augmentation technique is used for data augmentation. The results obtained are accurate and outperform the other existing methods.

## 3. Proposed Method

**Figure 2:**
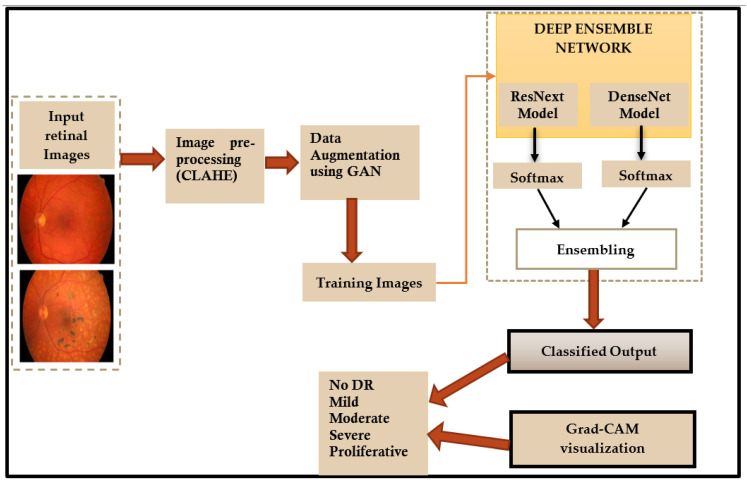
Flowchart of the proposed method.

In this paper, a deep learning ensemble model is used for detection and classification of diabetic retinopathy. The flowchart of the proposed method is shown in Figure 2.

The following sections present a detailed description of each step shown in the flowchart (Figure 2).

### 3.1. Input Retinal Images

The retinal images used are fundus images that are acquired under heterogeneous imaging conditions. The images are rated by a clinician indicating the level of diabetic retinopathy on a scale of 0 to 4 for five classes. The images with two classes are graded as DR and No DR. These images are used for testing and training the model.

### 3.2. Pre-Processing of Images

The images obtained are taken under different lighting conditions. These images need to be pre-processed before these can be used for model training. The retinal images have low contrast and therefore CLAHE is used for histogram equalization. The histogram equalization algorithm is as follows (Algorithm 1):
**Algorithm 1: Histogram equalization algorithm.****Input—Retinal images****Output—Preprocessed histogram equalized image****Method: CLAHE****Begin****For each image compute:**                 
$x_{k}\left(i,j\right)=\frac{\left(x_{kmax}-x_{kmin}\right)}{Ρ\left(x_{k}\right)}$**where **$x_{k}\left(i,j\right)$** is the pixel value at location **(i,j)**for the**$k_{th}$**image****End**

The pre-processed images obtained from this step are used as input to the proposed deep learning network.

### 3.3. Data Augmentation

**Figure 3:**
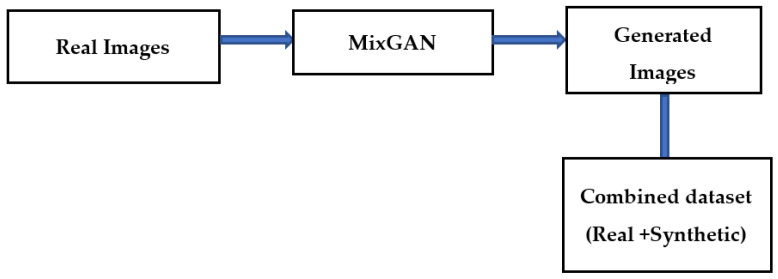
Data augmentation technique.

The bottleneck while developing an effective classification model for retinal images is the lack of adequate quantity of relevant class-specific data. Positive examples of disease conditions tend to be rare. Therefore, in this work, data augmentation is used to enhance the class-specific data. Many methods of data augmentation are available. Generative adversarial networks (GAN) are effective for data augmentation [20]. In GAN-based augmentation, the model is first trained using all the classes and thereafter fine tuning for rare classes occurs. In this paper, a MixGAN model is used for augmenting the data. This model performs better for data augmentation, since the model can capture mix-type data information as well as continuous data generation. Real images are given as input to the MixGAN model, and synthetic images are generated. Then the synthetically generated images are augmented in the available dataset. The process followed for generating the synthetic images is shown in Figure 3.

### 3.4. ResNeXt Model

**Figure 4:**
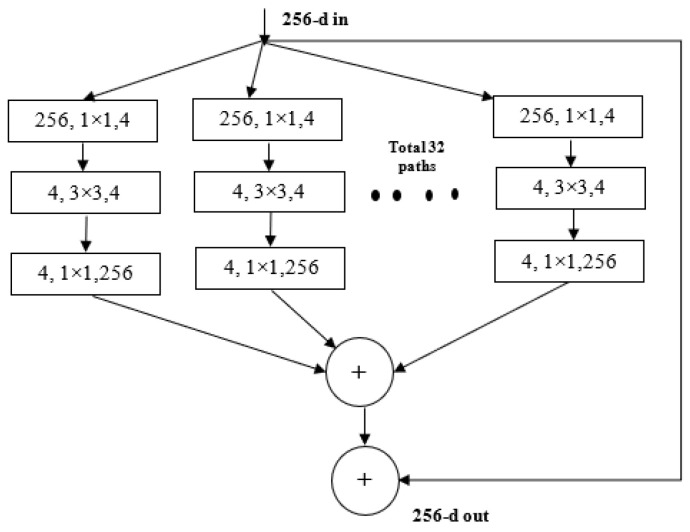
ResNeXt Block.

ResNeXt is a variant of ResNet it extends the concept of the residual network to the “split transform merge” technique. In this model, the convolutions are not performed on the full input feature map. Rather, the convolutions are performed on lower dimensional representations. These blocks are later merged after applying some convolutions. The ResNeXt block is shown in Figure 4.

### 3.5. DenseNet Model

**Figure 5:**
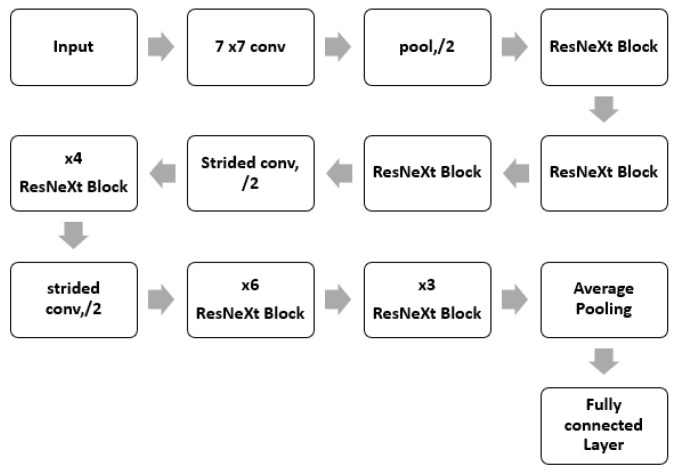
ResNeXt Architecture.

**Figure 6:**
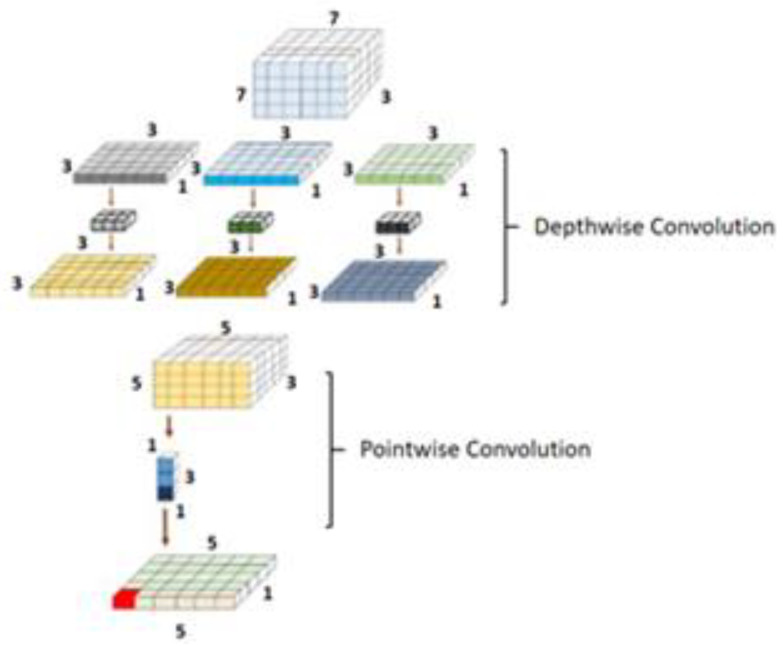
DenseNet block architecture.

The DenseNet comprises dense connections that outperform the simple ResNet and highway model. DenseNet solves the vanishing gradient problem by using the concatenation operation over the different blocks [21]. This network enables the maximum amount of information transfer amid the internal layers of the network. The complete architecture of the ResNeXt is shown in Figure 5. The dense block is shown in Figure 6. As it is observed, in this block, every layer is connected to all the proceeding layers. The output of a layer is derived from the outputs of all the previous layers. This dense connection overcomes the vanishing gradient problem. The network convergence becomes high, and the performance is also improved.

The architecture of the model used is shown in Table 1.

### 3.6. Ensemble Architecture

**Figure 7:**
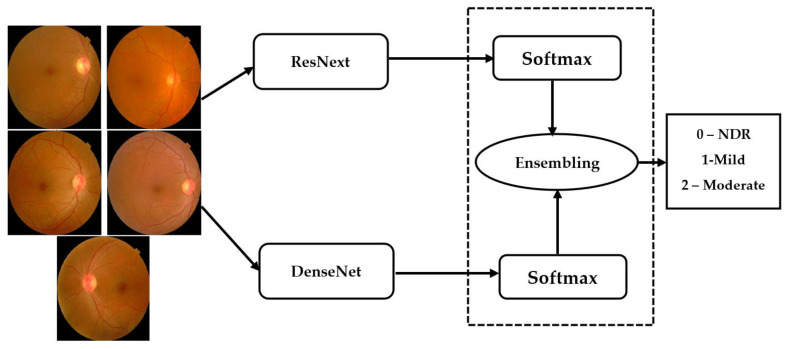
Ensembling architecture.

Ensemble networks combines several individual models to obtain a more efficient output. Deep ensemble learning models combine the advantages of both the deep learning models as well as the ensemble learning such that the final model has better generalization performance. In this work, ResNeXt v2 architecture is used [22]. This model is ensembled with the modified DenseNet, as shown in Figure 7.

The ensemble model inputs the outputs ($s_{c}^{i}$) obtained from the individual models (i ∈{1,…,n}) for a class c ∈C. The ensembling technique normalizes the output over each class. Thereafter, these are combined using the softmax layer to obtain the final output from the ensemble network.

(1) $$o_{i}=P\left(y=c|x,s_{i}\right)=\frac{e^{s_{i}^{c}}}{\sum_{c\;\in C}e^{s_{i}^{c}}}$$

(2) $$\arg max\;P\left(y=c|x\right)=\frac{e^{o_{i}}}{\sum_{i\;\in I}e^{o_{i}}}$$

Equation (1) performs the softmax over the class scores and Equation (2) performs a maximum a posteriori over the class outputs to compute the final class label. The loss function used is maximum probability cross entropy. The value of n is 2 and C is 5.

(a) Maximum probability-based cross entropy loss: For improving the training of the model, MPCE loss function is used. It reduces the back propagation error and makes the convergence fast. The mathematical formulation of MPCE is shown in Equation (3).

(3) $$f^{t}\left(W\right)=-\overset{m}{\underset{i=1}{\sum }}y_{i}^{\prime }log\left(y_{i}\right)=-\overset{m}{\underset{i=1}{\sum }}\left(y_{max}-y_{u}\right)\bar{y_{i}}log\left(y_{i}\right)\;$$

where, $y_{max}$ is the maximum amongst *m* classes with the true class being the $u_{th}$ class. The *u_th_* coordinated $\bar{y}$ is 1, $\bar{y}$ is the vector of real classes. And $y_{i}^{\prime }$ is the $i_{th}$ coordinate of the vector $y^{\prime }$.

### 3.7. Model Training

The training of a deep neural network (DNN) requires two major components, i.e., a loss function or training objective and an optimization algorithm. Loss function is used to estimate the loss of the model by comparing target value with predicted value of DNN. The optimization algorithm minimizes the loss function by updating the weights of the network. We have used categorical cross entropy loss function available in keras library. It is a softmax activation plus a cross entropy loss. It is used for multi-class classification problems to output the probabilities over the n number of classes for each image. In medical datasets, an imbalanced dataset is a frequent problem for building a deep learning model due to lack of a sufficient amount of the training data or uneven class distribution within the dataset. For handling an imbalanced dataset problem, we have used the strategy of data augmentation, as discussed above. For efficient memory utilization, we use mini-batches, in which a small subset of all samples is propagated through the network during training. After relevant experiments, the number of samples per batch was set to eight. The epoch is the model hyper parameter which is defined as the number of times the network will be trained over the entire training dataset. The performance of the models is assessed with different evaluation metrics such as F1-score, Precision, Validation Accuracy, Sensitivity, Specificity, etc., which is detailed in the Results section.

### 3.8. Rectified Adam Optimization

In this paper, a rectified Adam optimization algorithm is used. It is a variant of the Adam optimizer that introduces a term to rectify the variance of the adaptive learning rate. It seeks to tackle the bad convergence problem suffered by the Adam optimizer.

### 3.9. Grad-CAM Visualization

The gradient-based class activation map (Grad-CAM) is a class-discriminative localization map which highlights the relevant regions of image by computing gradient of class score y^c^ for class c in reference to feature map activations A^k^ of a convolutional layer, i.e., ∂y^c^/∂A^k^. These gradients flows back and are then global-average-pooled to obtain the importance of weights of neurons, i.e., a^c^_k_ [17].

(4) $$\propto_{k}^{c}=\overset{︷}{\frac{1}{Z}\underset{i}{\sum }\underset{j}{\sum }}$$

Grad-CAM is basically a weighted combination of forward activation maps followed by the ReLU operation, as follows:

(5) $$L_{Grad-CAM}^{c}=ReLU\left(\underset{k}{\sum }\propto_{k}^{c}A^{k}\right)$$

**Figure 8:**
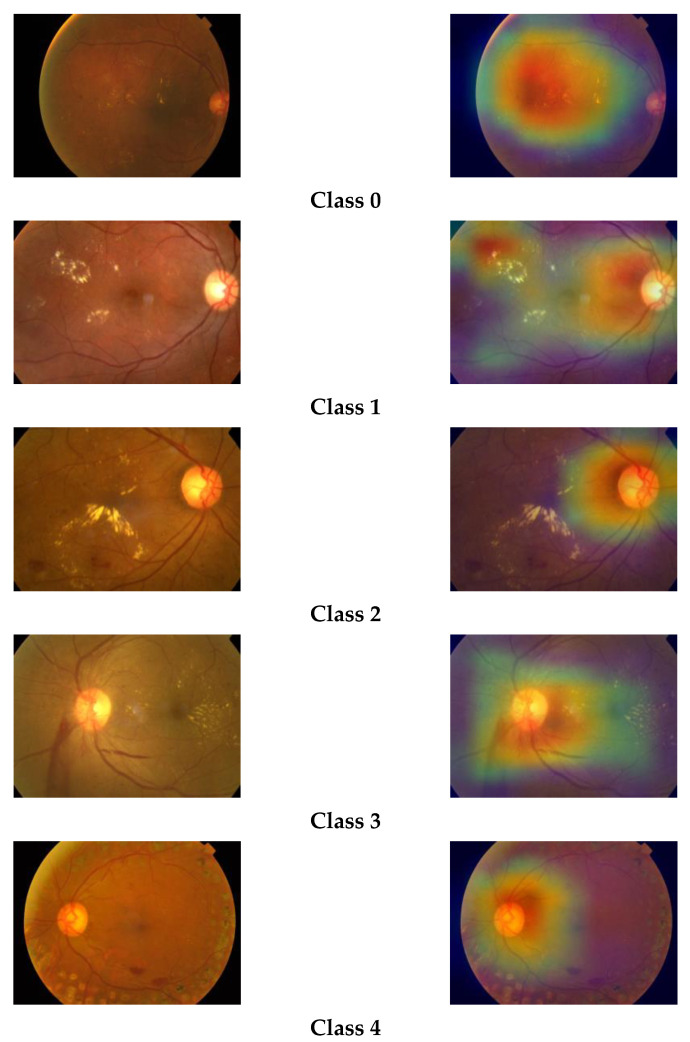
Grad-CAM visualizations of retinal images.

We have plotted the Grad-CAM visualization heatmap of sample test images of the three classes predicted by our model. The Grad-CAM visualization heatmap highlights the relevant regions in the image, which the final convolution layer of the model uses to discriminate among different classes. We can observe that the Grad-CAM visualization heatmap produces different highlighting patterns for normal and DR images. The class activation map of a normal eye highlights the full image, focusing on the middle region, whereas, in the case of DR images, the upper region of the image is highlighted with greater density. The highlighted part in the class activation map is the important region in the image, used by the model for predicting the concept. The gradcam visualizations for different classes are shown in Figure 8.

## 4. Results and Discussion

The experiments are conducted in Python with GPU acceleration. The keras module is used for implementing the deep learning model.

### 4.1. Dataset Used

#### 4.1.1. Two Class Databases

The first dataset used is DIARETDB1 database which is available publicly, for use by researchers. This dataset is used for two class detection, i.e., DR and no DR [23]. The dataset contains ground truth data marked by experts. It comprises of 89 color fundus images; the dataset has two gradings: Diabetic Retinopathy (DR) and No Diabetic Retinopathy (NDR).

#### 4.1.2. APTOS 2019

The data are collected from the APTOS 2019 diabetic retinopathy dataset. The dataset is available on Kaggle [24]. The dataset is collected by Aravind Eye Hospital in India’s rural areas for the development of automated tools for DR detection. The dataset comprises a large set of retina images taken using fundus photography under a variety of imaging conditions. Each image is rated for the severity of diabetic retinopathy on a scale of 0 to 4. The images are either No DR, Mild, Moderate, severe or Proliferative DR. The grading from 1–3 is conducted based on the frequency, location, and severity. The distribution of training images is shown in Table 2.

**Figure 9:**
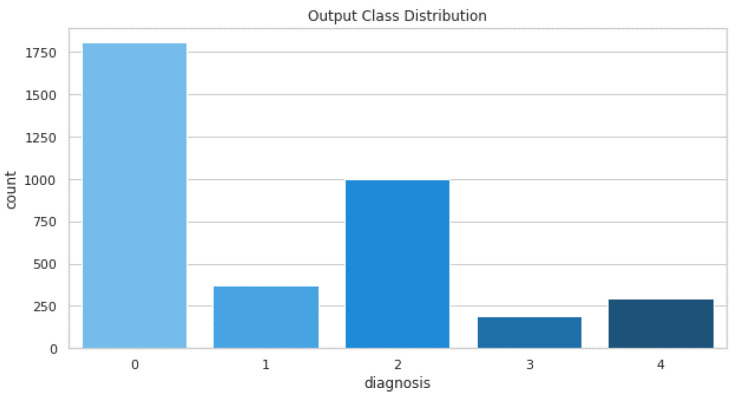
Training images distribution.

The dataset comprises a total of 3662 training images and 1928 testing images. The distribution of these images is shown in Figure 9.

**Figure 10:**
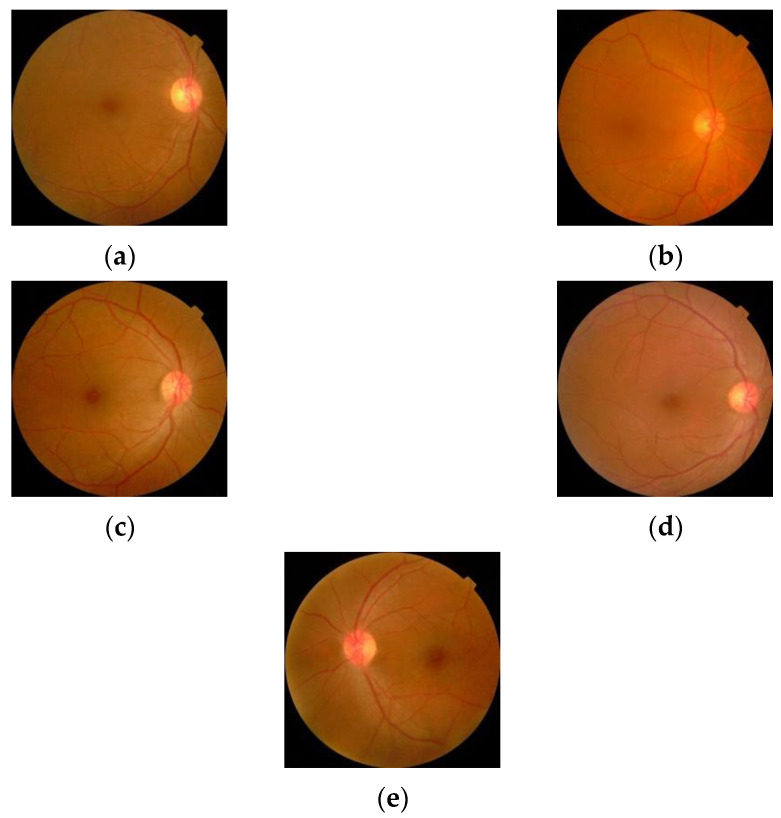
Retinal images for (**a**) Normal (**b**) Mild DR (**c**) Moderate DR (**d**) Severe DR (**e**) Proliferative DR.

The sample images from the database are shown in Figure 10.

The performance of the proposed method is evaluated using the various evaluation metrics. The following metrics are used:

- Precision is defined as the number of misclassifications. This can be computed by

(6) $$\mathrm{Precision}=\frac{TP}{TP+FP}$$

- Recall is a measure of the actual positives a model computes. The formula for computing the recall is shown in Equation (6):

(7) $$\mathrm{Recall}=\frac{TP}{TP+FN}$$

- The overall accuracy is also computed using

(8) $$\mathrm{Overall}\;\mathrm{Accuracy}=\frac{TP+TN}{TP+TN+FP+FN}$$

where TP, FP and FN represent the true positive, false positive and false negative, respectively.

#### 4.1.3. Two Class Detection

The experiments were also conducted for two class detections. The two classes are normal and abnormal. The confusion matrix for the two classes is shown in Table 3. The 89 images were manually assigned into categories representing the progressive states of retinopathy. Using the categories, the images were divided into the representative training (28 images) and test sets (61 images).

#### 4.1.4. Five Class Detection

The dataset comprises of five classes of DR images that are represented by the numbers 0 to 4.

0 No DR (NDR)
1 Mild
2 Moderate
3 Severe
4 Proliferative DR (PDR)

The testing was conducted on 733 testing images from different grades. The grade-wise distribution of images is shown in Table 4.

The confusion matrix for five classes is shown in Table 5.

The precision, recall and accuracy of the method is calculated and compared with other state-of-the-art methods.

The results obtained for different methods for two classes and five classes are shown in Table 6 and Table 7, respectively.

The results show that the proposed method performs better than the existing methods for both two classes and five classes. For five classes, the model accuracy is less than that for two classes. This cam be further improved by using a more balanced dataset for five classes. Thus, the limitation of the model is the imbalanced and limited medical data for five classes.

## 5. Conclusions and Future Work

Diabetic retinopathy is affecting millions of individuals worldwide. Early detection of DR aids in the treatment of the affected individuals. In this paper, a deep ensembled model for detection and classification of DR from retinal fundus images is presented. The proposed method follows an ensemble approach using ResNeXt and a modified DenseNet deep learning model. The model is trained on the APTOS dataset that consists of five classes of images. Another dataset, used for two classes, is DIARET DB1. The input images are pre-processed to equalize the histogram using the CLAHE method. The class imbalance is handled using MixGAN data augmentation. The augmented dataset is used to train the model. The performance of the proposed method is compared with other state-of-the-art methods. The results show that the proposed method outperforms the other methods. In future, it will be beneficial if the efficiency of the proposed model is increased. In addition, the classification of DR in various classes can be improved. A device can be developed using the proposed method that can completely automate the disease detection and classification.

## Figures and Tables

**Table 1:** Architecture of DenseNet model.

Layer Type	Output Shape	#Parameters	Kernel Size	Dropout	#Filters
Input	(224,224,3)	0	-	0	-
Conv2d ×2(RAdam)	(224,224,16)	448	3 × 3	0	4
Maxpooling 2d	(112,112,16)	0	-	0	-
Seperable Conv2d ×2(ReLU)	(112,112,32)	668	3 × 3	0	32
Batch Normalization	(112,112,32)	128	-	0	-
Maxpooling 2d	(56,56,32)	0	-	0	-
Seperable Conv2d ×2(ReLU)	(56,56,64)	1344	3 × 3	0	64
Batch Normalization	(56,56,64)	256	-	0	-
Maxpooling 2d	(28,28,64)	0	-	0.2	-
Seperable Conv2d ×2(ReLU)	(28,28,128)	2400	3 × 3	0	128
Batch Normalization	(28,28,128)	512	-	0	-
Maxpooling 2d	(14,14,128)	0	-	0.2	-
Seperable Conv2d ×2(ReLU)	(14,14,256)	4736	3 × 3	0	256
Batch Normalization	(14,14,256)	1024	-	0	-
Maxpooling 2d	(7,7,256)	0	-	0.2	-
Seperable Conv2d ×2(ReLU)	(7,7,256)	8896	3 × 3	0	256
Batch Normalization	(3,3,256)	1024	-	0	-
Maxpooling 2d	(3,3,256)	0	-	0.2	-
Seperable Conv2d ×2(ReLU)	(3,3,512)	17,664	3 × 3	0	512
Batch Normalization	(3,3,512)	2048	-	0	-
Maxpooling 2d	(1,1,512)	0	-	0.2	-
FC1(ReLU)	(512)	262,656		0.7	512
FC2(ReLU)	(128)	65,664		0.5	128
FC3(ReLU)	(64)	8256		0.3	64
FC4(ReLU)	(32)	2080		0.2	32
FC5(ReLU)	(5)	165		0	5
Total Params: 1,021,925 Trainable params: 1,019,429 Non-trainable params: 2496					

**Table 2:** Distribution of training images.

Grade	No. of Images (Training)
0—No DR	1805
1—Mild	370
2—Moderate	999
3—Severe	193
4—Proliferative	295

**Table 3:** Confusion Matrix for two classes.

*Predicted*			
***Actual***		NDR	DR
	NDR	**29**	2
	DR	3	**27**

**Table 4:** Testing image distribution.

Grade	Testing Images
0—No DR	353
1—Mild	87
2—Moderate	205
3—Severe	40
4—Proliferative	48
Total	733

**Table 5:** Confusion matrix for five classes.

		*Predicted*				
***Actual***		NDR	Mild	Moderate	Severe	PDR
	NDR	**313**	16	8	10	6
	Mild	5	**71**	6	4	1
	Moderate	8	8	**179**	7	3
	Severe	1	3	3	**25**	8
	PDR	1	1	2	1	**43**

**Table 6:** Results comparison, two classes.

Method	Precision	Recall	Accuracy
DRISTI (VGG16 + Capsule) [25]	0.96	0.96	96.24
EfficientNet-B3 [26]	0.95	0.96	96.07
Resnet50 + Capsule [25]	0.94	0.93	95.54
EDLDR (Proposed Method)	0.97	0.97	96.98

**Table 7:** Results comparison, five classes.

Method	Precision	Recall	Accuracy
DRISTI (VGG16 + Capsule) [25]	0.91	0.88	82.06
EfficientNet-B3 [26]	0.59	0.66	84.86
Resnet50 + Capsule [25]	0.59	0.69	76.80
EDLDR (Proposed Method)	0.76	0.82	86.08

## Data Availability

Publicly Available (https://www.kaggle.com/c/aptos2019-blindness-detection).
